# Evaluation of Change in Center of Pressure During Perturbation of Balance Including Blindfolding in Healthy Dogs

**DOI:** 10.3390/ani15121790

**Published:** 2025-06-18

**Authors:** Hayley Hall, Richard B. Evans, Makayla Balogh, Wanda J. Gordon-Evans

**Affiliations:** 1 Veterinary Medical Center, College of Veterinary Medicine, University of Minnesota, St. Paul, MN 55108, USA; 2 Clinical and Translational Science Institute, University of Minnesota, Minneapolis, MN 55414, USA

**Keywords:** postural control, physical therapy, rehabilitation, center of pressure

## Abstract

Physiotherapy aims to improve postural dysfunction, often using balance exercises. The effects of rehabilitation-based balance exercises on postural control in dogs remains understudied. This study aimed to assess the impact of specific balancing exercises and blindfolding on center of pressure parameters in healthy dogs. Thirteen healthy adult dogs participated in the study. Center of pressure parameters were measured using a pressure platform during quiet standing, external perturbation, head turn, and blindfolded trials. External perturbation significantly increased the area of the elliptical sway, cranio-caudal excursion, and right–left excursion. Head turning led to large effect sizes, but no significant differences compared to quiet standing. Blindfolding led to medium to large effect sizes, though no significant differences were observed. The study confirmed that external perturbation challenges postural stability, resulting in increased sway. Head turning induces sway but may require further training or different methodologies for reliable outcomes. Blindfolding increased sway but was not statistically different. These findings underscore the potential use of perturbation-based exercises in canine rehabilitation to improve balance, while also highlighting the need for further studies to standardize balance challenges and explore the effects in dogs with orthopedic or neurological conditions.

## Simple Summary

Postural control and rehabilitation are emerging areas of study in veterinary medicine. In dogs, the effects of rehabilitation-based balance exercises and vision loss on postural control remain understudied. The present study evaluated these effects using changes in center of pressure parameters, finding that external perturbation significantly challenged postural stability, whereas there were no statistical differences in head turn or blindfolded animals. Further research is needed to understand the long-term effects of balance exercises and their application in dogs with pre-existing orthopedic and neurological conditions.

## 1. Introduction

Postural control is the act of maintaining, achieving, and restoring the body to a state of balance through compensatory or anticipatory strategies [1,2]. Postural stability involves controlling the center of mass (COM) within the base of support [2,3]. COM is the point at the center of total body mass. The vertical projection of the COM is the center of gravity (COG) [3]. In healthy patients, if the COM or COG shifts outside the base of support (BOS), then compensatory actions, such as protective stepping or activating stabilizing muscle groups, help to prevent falls [1,2,4]. Maintaining postural control relies on the integration of visual, somatosensory, and vestibular systems, followed by a musculoskeletal response [1,2]. Dysfunction or degeneration of any of these systems may impair postural regulation, thus increasing the risk for fall or injury.

Disturbances in COM have been studied in both human and veterinary literature through the evaluation of center of pressure (COP) [2,5,6,7,8,9,10,11,12,13,14,15,16,17,18,19,20,21,22,23]. COP is the center of the distribution of the total force applied to the supporting surface [3,24]. In other words, as COM moves toward the periphery of the base, COP is a way of measuring the pressure to correct toward stability [24]. It is measured using the ground reaction forces of the feet over the surface area. The COP is continuously moving to keep the COM within the BOS causing a sway when standing [3].

In humans, postural dysfunction is often associated with pain avoidance or compensation for deficits in strength or coordination and can be measured during gait or while standing [25,26,27]. In dogs, pressure walkways have been used to measure COP in standing and during gait as well [14].

Recent veterinary studies have shown that sway increases in dogs with lameness due to elbow dysplasia, cranial cruciate ligament disease, or aging [2,6,7,9,11]. Change in motion can be measured at the level of the paw or body. Paw and body COP have been evaluated in dogs with elbow arthritis [16], elbow dysplasia, and cranial cruciate ligament rupture [15]. Paw COP has been evaluated in dogs with elbow dysplasia and compared between healthy and dogs with osteoarthritis [17,18]. Juvenile dogs that are later diagnosed with hip dysplasia exhibit elevated paw COP parameters during growth compared to age-matched healthy individuals [19]. Similarly in healthy chondrodystrophoid dogs and dogs with spinal cord injury while walking on an instrumented treadmill, COP was increased [20,28].

Rehabilitation and physiotherapy are effective in restoring normal movement patterns, often with the goal of improving postural dysfunction [2]. In humans, perturbation-based training for those prone to instability has been shown to significantly reduce fall risks [29,30]. In animals, rehabilitation is commonly sought for orthopedic and neurologic injuries with balancing exercises often recommended to improve postural response. Measuring how much the animal sways may be a tool for assessing response to treatment or for assessing how effective the exercises are at testing postural control.

Research on external perturbations affecting balance control is more extensive in human medicine, with studies focusing on variable surfaces, mechanical platforms, single leg stances, and waist pulls [4,29,31,32,33,34,35]. In veterinary medicine, external perturbation has been assessed on a motorized balance board, showing that amplitude affected COP more than speed [9]. An additional study evaluated static and dynamic body COP at two time points in healthy dogs and found reliability in a balance platform to assess static posturography but that there were training effects from session to session during dynamic posturography [36]. While motorized platforms are excellent methods to regulate external perturbation, they require specialized equipment that is not widely accessible. Manual external perturbation, head turning, and blindfolding are more accessible and are exercises based on rehabilitation. It is assumed that perturbing balance, helps to facilitate increased ability to accommodate balance disruptions and improve gait and standing control, but the amount of perturbation of these common exercises has not been investigated.

Evaluation of visual input on postural control has been performed in previous equine [37] and human literature [38,39]. A recent study has evaluated this in canines, finding that senior dogs had increased craniocaudal sway under the blindfolded condition compared to adult dogs, while adult dogs exhibited reduced sway under the blindfolded condition [10]. However, there is still a paucity of research assessing the impact of visual input in dogs.

Therefore, this study aimed to assess the changes in COP during specific balancing exercises and blindfolding in healthy dogs using a four-way (standing, blindfolded, direct perturbation, and head turn) crossover study. We hypothesized that the COP would increase in distance and area over quiet standing with all testing conditions.

## 2. Materials and Methods

### 2.1. Ethics

Data were obtained from voluntary and healthy participants with permission from their owners. Positive reinforcement was highly utilized to improve compliance, and trials were discontinued if dogs experienced discomfort, stress, or were intolerant. The study procedure was approved by the Institutional Animal Care and Use Committee (2407-42227A).

### 2.2. Participants and Inclusion Criteria

The sample size was calculated a priori using Shaheen et al. where the right–left (RL) movement was 2.8 cm with a standard deviation (SD) of 1.1 cm [13]. Using a difference of 1 cm from normal as clinically relevant, 12 dogs would be necessary. Using Mondino et al., the sample size was similar, with 13 dogs needed. Dogs were excluded if they had pre-existing conditions that would affect balance, such as neurological disease, orthopedic disease, and visual or auditory deficits. Each dog underwent an orthopedic and neurological examination to evaluate for underlying disease. Only dogs aged one year or older, without known pre-existing conditions, and with unremarkable orthopedic and neurological examinations were included in the study.

### 2.3. Procedure and Equipment

Data were collected with a pressure-sensitive walkway (Strideway; Tekscan, South Boston, United States). Data was collected on one tile (2.1 × 2.1 ft) with embedded pressure sensors called ‘sensels’ at a density of 3.88 sensels/cm^2^. The pressure-sensitive walkway was connected to a dedicated computer with a specific software (Strideway Research: Tekscan, Version 7.8.1). The SAM^TM^ software module for sway analysis was utilized [40].

Each sensel of the Strideway produces a pressure output recording that is coordinated to the calibrated parameters. The calibration files were formed based on weight. Prior to each trial, the walkway was calibrated as directed by the company. The walkway plate detected pressures at a sampling rate of 60 Hz for 5 s. The software calculates the center of force (COF) and measures the area of the elliptical region where the COF spent 95% of its time, total distance traveled, cranial-caudal (CC) excursion, right–left excursion, and variance from the COF. Although measuring the COF, past studies have used the measurements synonymously with COP [41,42].

The dogs were recorded for 3–5 valid trials based on tolerance, with 5 trials being the goal, for 5 s intervals. The dogs were evaluated under four different conditions: quiet standing, external perturbation, head turn, and blindfolded. To be considered valid, the dog had to tolerate the testing condition without movement of head, limbs, body, or tail, except in the head turn activity, as evaluated by 2 investigators actively monitoring for deviations.

All measurements were stopped at any time if the dog was uncooperative, tired, or painful. Five-minute rests were allowed between trials as needed. All dogs were bribed with treats and praise for voluntary and fear-free cooperation.

**Figure 1:**
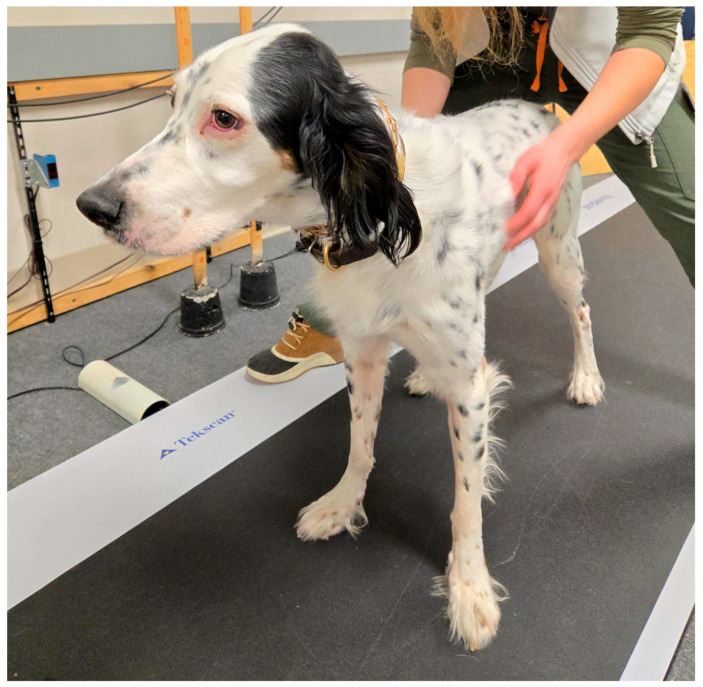
Experimental setup for a dog undergoing external perturbation. The dog is standing in a neutral position. One investigator provides external perturbation from behind with a flat palm, alternating from side to side on the dog, while a second investigator holds attention at the front of the dog. The recording computer is located to the right of the dog.

For quiet standing trials, dogs were walked onto the pressure plate and asked to stand with the head forward and all four limbs square on the plate with focus on the handler using a toy, sound, or treat. Treats were withheld until all trials were completed if dogs were overstimulated by them. External perturbation was then applied by the handler, with gentle pressure applied to the right mid-chest then the left mid-chest just caudal to the scapula for the duration of the test starting at the same time as the data recording (Figure 1). Because algometry failed to provide consistent perturbation without causing a flinch, hand pressure was utilized. Visual assessment of body movement without compensatory stepping was used to judge the pressure applied.

Trials of head turns were obtained using treats at the level of the nose to guide the head to the right and to the left. Trials were considered valid if the dog did not change foot position but turned their head past 90 degrees in both directions during the 5 s recorded.

Blindfolded trials were performed by walking the visual dog onto the pressure plate and asking them to stand with their head forward and limbs square. A soft cloth blindfold was then placed over the eyes and the trial recorded. Validity criteria were identical to the standing condition.

The software generated the following outcome measures: total area where the COP spent 95% of time (cm^2^), distance of the path of the COP (cm), the standard deviation (variation) of the COP from frame to frame (cm), cranial-caudal excursion (cm), and left-right excursion (cm) [5,7,8,9,10,11,13,15,16,17,18,19,20,21,28,36,40,43,44,45,46,47,48].

### 2.4. Statistical Analysis

The data analysis was in three parts. First, descriptive statistics (means, standard deviations, and medians) and histograms were used to check the data for spurious observations and data distributions, as well as summarize the data. Second, inferences were performed for all testing conditions and outcomes, (all were continuous, measured in cm or cm^2^), using the Wilcoxon signed-rank test, which is a conservative, robust test for matched pairs. The Bonferroni correction was used to control for Type I error inflation arising from calculating 15 statistical tests, so statistical significance was set at *p* < 0.003. Finally, the magnitude of change was reported using Cohen’s *d* effect size for each testing condition. Cohen’s *d* represents the number of standard deviations difference between means and is independent of sample size. A Cohen’s *d* of 0.5 is a moderate effect size, and a Cohen’s *d* of 1.0 is considered a large effect size [49].

## 3. Results

Thirteen dogs met the inclusion criteria for this study and were enrolled in the sway analysis evaluation. Thirteen dogs were examined, and no dogs were excluded based on their orthopedic and neurological examinations. There were 9 females (8 spayed and 1 intact) and 4 males (4 neutered and 0 intact). The breeds of the dogs were as follows: one Rat Terrier, three Labrador Retrievers, five mixed-breed dogs, two King Cavalier Charles Spaniels, one Great Dane, and one Standard Poodle. The mean body weight (range, SD) was 22 kg (6.3 kg to 44.5 kg, 10.77). The mean age (range, SD) was 3.4 years (1 year to 8 years, 2.11). The mean body condition score (range, SD) on a scale of 9 was 4.8 (4 to 6, 0.58).

**Figure 2:**
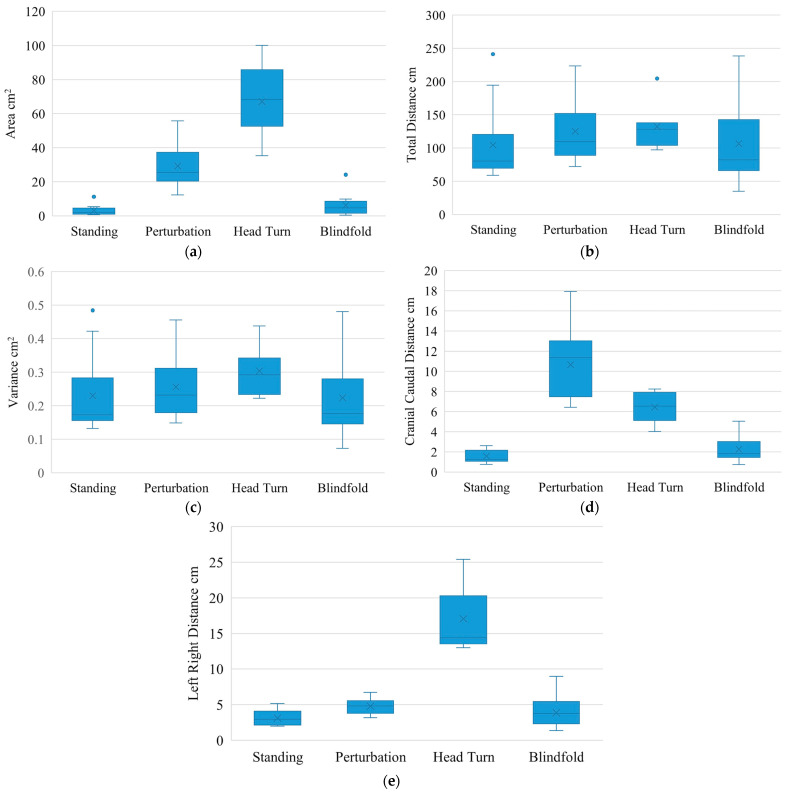
Box and whisker plots for the COP parameters under each condition: (**a**) Area of the ellipse where COP spent 95% of its time (cm^2^) for each condition; (**b**) Total distance traveled by COP (cm) for each condition; (**c**) Variance of the COP (cm^2^) for each condition; (**d**) Cranial-Caudal excursion (cm) for each condition; (**e**) left–right excursion (cm) for each condition.

All dogs completed 5 trials under the standing condition. Under the external perturbation condition 12 dogs completed 5 trials and 1 dog completed 3 trials. Under the head turn condition 6 dogs completed 5 trials, 1 dog completed 3 trials, and 6 dogs were unable to perform a head turn successfully. These 6 dogs would all step back the forelimb ipsilateral to the head turn. Under the blindfolding condition, 10 dogs completed 5 trials, and 3 dogs completed 3 trials. COP parameters for each dog and data histograms are found in the Supplementary Data. Figure 2 shows the box and whisker plots for the parameters under each condition.

Compared to quiet standing, all measured conditions led to an increase in sway parameters. Significant changes were only noted with external perturbation in the area of ellipse (*p* = 0.0002), cranial-caudal distance (*p* = 0.0002), and right–left distance (*p* = 0.0005). The effect size, as evaluated by Cohen’s *d*, was large for all the aforementioned parameters and was greater for difference in the cranio-caudal distance (Cohen’s *d* = 2.5) than in the right–left distance (Cohen’s *d* = 1.3) (Table 1).

There was no significant difference in measured parameters for the head turning condition compared to quiet standing. However, all the effect sizes were large, with Cohen’s *d* ≥ 2.0.

In the blindfolded condition, the smallest overall difference was noted, and the effect size was medium in the right–left distance and large in the cranio-caudal distance [49].

## 4. Discussion

This study evaluated the changes in COP parameters in healthy dogs during blindfolding and specific balance exercises including external perturbation and head turns. We hypothesized that the COP would increase in distance and area over quiet standing with all testing conditions. However, this proved to be only partially true.

At quiet standing, dogs exhibited greater RL excursion compared to CC excursion, suggesting less stability in the RL direction. This finding aligns with previous canine studies [15,16], although these studies evaluated either the forelimbs or hindlimbs which is a technique that has not yet been validated in dogs. The greater length of the base of support in the CC axis may contribute to this pattern, as humans, with a wider base of support in the RL axis, typically exhibit more sway in the anterior–posterior direction [47,50]. This contrasts with a previous study that reported significantly greater CC excursion in dogs during static measurement [9]. However, further investigation is warranted due to the conflicting literature in canines.

During external perturbation, there was a significant increase in CC excursion, RL excursion, and the area of ellipse. Previous research using a Posturomed balance platform found that the mean area of ellipse was significantly higher under Dy- and Dx- conditions compared to static measurement, and that this was the most reliable measure between two different time periods [36]. An additional study showed that external perturbation on a motorized platform leads to significant increases in RL excursion, CC excursion, support surface (area of ellipse containing 90% of the points of the COP trajectory), and length as a function of surface, without significant differences in total distance traveled, and COP speed [9]. This is consistent with findings in the present study regarding the external perturbation condition; however, a limitation of this study was a lack of standardization for right to left pressure, which may have introduced human error in terms of force and location of the perturbations. Future research may consider an accessible standardization method utilizing hand pressure sensors or furthering research using previously evaluated platforms [9,36].

During head turning, there was no statistical difference in any measured COP parameter, although large effect sizes were noted for each parameter in the dogs that completed the test. This suggests that while head turning does alter sway and challenge balance, it may not serve as a reliable outcome measure in this context. Changes in head position have been used in human studies to evaluate concussion patients [51], as head position can alter proprioception and muscle activation, affecting balance [52]. In this canine population, almost all dogs stepped a forelimb backward instead of head turning. Despite attempts at training, six dogs were unable to successfully complete the minimum 3 trials without stepping. All dogs were naïve to head turning exercises prior to the study, and this was challenging to train in a short time period allotted for data collection. Although based on the effect size head turns are a good balance exercise, future studies should consider implementing a longer training period, using a canine population that have experienced head turning in physiotherapy, or exploring alternative head movements when evaluating head position in dogs.

Finally, during blindfolded trials, there were no significant differences in any COP parameters compared to quiet standing. A recent study evaluated the impact of visual loss in canines, finding that under the eyes closed condition, healthy adult dogs had a significant reduction in craniocaudal displacement with no effect on RL displacement, total sway length, or support surface, and senior dogs did not significantly change in any parameter when eyes were closed [10]. Similar to the senior dogs, the present study showed no statistical difference from standing; however, the means of all parameters were increased in the blindfolded group [10]. This may be due to low sample size and high variation in the dogs included. For example, age varied from 1 to 8 years old. There was only 1 dog considered senior as defined by Lutonsky et al. [10], and this dog’s individual data was close to the overall mean for all sway variables.

Studies in horses have shown that blindfolding increases sway in both the CC and RL directions [37] and human studies also report increased sway in eyes-closed conditions [47,53]. Dogs in this study are similar to humans and horses in this regard, although lack of significant differences may be due to extra stability provided by four limbs on the ground and digitation.

Another possible reason that stability was not statistically different in blindfolded dogs is behavioral. Convincing dogs to make an effort to be as still as possible is difficult, and it is possible that they tried harder to be still when blindfolded. Perhaps if the efforts were equivalent, standing would be more stable, allowing for a significant difference. It is still unknown if blindfolding would be a useful method of determining postural dysfunction in neurologically or orthopedically diseased dogs. Blindfolds were well tolerated by this study group and further studies could explore these questions.

This study has several limitations. The first is the measurement of COF rather than COP. The COF is directly related to the COP and takes into account multiple directional forces [54]. In this context, COF and COP would have the same distance measurements although COP is the more commonly used term. COP is commonly reported even when using the same software and platform, thus it is the term used in this study [41,42].

Furthermore, there is no set gold standard for measuring COP. Trial number, sample frequency and length of trial acquisition is highly variable [5,6,7,8,9,10,11,13,14]. Although validation has been attempted, the methods are rarely repeated identically or with identical equipment [6,8,13]. In this study, a minimum of three trials was acceptable with five trials as the goal with a midrange sample frequency, and 5 s trial acquisition time using equipment that has been validated for sway measurement in humans and for pressure platform gait analysis in dogs [41,42,55]. The literature varies from 3 to 10 trials collected so the decision was arbitrary based on gait analysis experience [5,6,7,8,9,10,11,13,14]. In addition, frequency ranges from 1 frame per second for 10 s [7] to 100 frames per second with recording times ranging from 5 s to 1 min [5,6,7,8,9,10,11,13,14]. In one trial, 10 trials were collected for 8 s at 67 frames per second, but only 70 frames were used in each trial amounting to just over 1 s of time [6], and another recorded for 1 min and chose 20 s stretch in 3 trials [9]. The parameters used in this trial aimed to limit variation and maximize positive dog cooperation.

Secondly, there was a lack of standardization of the external perturbation trials. It was initially attempted to use an algometer to measure perturbation pressure, but this was unsuccessful as it induced a flinch. Perturbation was then induced by an investigator in an effort to push hard enough to cause a weight shift but not hard enough to make the dog step. This was subjective and hand proximity may have provided other proprioceptive clues or the ability to anticipate the perturbation.

We had multiple researchers monitoring body and head movements during the data collection to eliminate invalid trials; however, even small head movements contribute to variation. Treats, praise, and noise were used to attract and keep attention forward. With a larger study duration, more time could be devoted to an acclimation and training period. In addition, variation was limited by averaging a minimum of 3 trials and the change compared to standing was compared rather than the absolute measurements for each type of perturbation.

Additional limitations included a predominance of large breed dogs and an overall small sample size. Large breed dogs may not be representative of the total population and different morphologies may play a role in variation, especially as dogs adjust to perturbation. The sample size was calculated a priori and appropriately showed significance for multiple parameters so although it is small, it is arguably big enough to address the scientific question for most parameters. Future studies should base the sample size on the specific parameter of interest and diversity in dog size, age and breed should be limited depending on the scientific question. For instance, senior dogs may be excluded from blindfolding study cohorts.

## 5. Conclusions

Understanding how specific balance challenges affect postural control in healthy dogs can help inform rehabilitation protocols for those recovering from orthopedic or neurological conditions. Given that perturbation exercises have been shown to reduce falls in humans [29], perturbation exercises are likely useful for dogs in a similar manner. This study shows that external perturbation and head positioning challenge postural stability in dogs and are therefore likely helpful when integrated into physiotherapy programs. Head turns induced greater sway but were an unreliable study measurement. Blindfolding did not induce great change in healthy dogs but could be useful in evaluating neurological or orthopedic diseases. The reliability and validity of this study should be specifically evaluated as outcome measures in future studies for different disease processes and as a measure of improvement for animals in rehabilitation programs. Studies exploring the effects of balance exercises on COP parameters in dogs with orthopedic or neurological conditions, as well as the long-term impact of perturbation-based training on balance recovery or injury prevention, would provide valuable insights for future veterinary care.

## Figures and Tables

**Table 1:** Differences in COP parameters of measured conditions (perturbation-standing, head turn–standing, and blindfolding–standing) as compared to quiet standing. Significant *p*-values (*p* < 0.003) are starred (*). Cohen’s *d* is reported.

	Standing		Perturbation				Head Turn				Blindfold			
Outcome	Mean (SD)	Median	Mean Difference (SD)	Median Difference	*p*-Value	*d*	Mean Difference (SD)	Median Difference	*p*-Value	*d*	Mean Difference (SD)	Median Difference	*p*-Value	*d*
**Area (cm^2^)**	3.2 (2.9)	2.0	26.1 (12.9)	22.6	0.0002 *	2.0	64.1 (24.0)	64.5	0.0156	2.7	3.0 (4.2)	2.2	0.0266	0.7
**Distance (cm)**	104.5 (54.9)	80.5	20.7 (24.9)	26.2	0.0171	0.8	48.6 (23.6)	46.9	0.0156	2.1	2.0 (22.6)	1.7	0.6848	0.1
**Variance (cm^2^)**	0.2 (0.1)	0.2	0.0 (0.1)	0.0	0.0803	0.5	0.1 (0.1)	0.1	0.0156	2.0	0.0 (0.0)	0.0	0.8926	−0.1
**Cranial Caudal Distance (cm)**	1.6 (0.7)	1.3	9.1 (3.6)	10.2	0.0002 *	2.5	5.1 (1.8)	5.8	0.0156	2.8	0.7 (0.8)	0.6	0.0105	0.8
**Left Right Distance (cm)**	3.1 (1.1)	2.9	1.7 (1.0)	1.3	0.0005 *	1.6	14.3 (5.1)	12.4	0.0156	2.8	0.7 (1.5)	0.3	0.1272	0.5

## Data Availability

The original contributions presented in this study are included in the article/Supplementary Material. Further inquiries can be directed to the corresponding author.

## Supplementary Materials

The following supporting information can be downloaded at: https://www.mdpi.com/article/10.3390/ani15121790/s1, Table S1: COP parameter values for each participant for each trial performed. Figure S1: Histograms of COP parameters for each tested condition and for the differences in COP parameters between each tested condition as compared to quiet standing.

## Abbreviations

The following abbreviations are used in this manuscript:

COF	Center of Force
COP	Center of Pressure
COM	Center of Mass
COG	Center of Gravity
BOS	Base of Support
RL	Right–Left
CC	Cranio-Caudal
SD	Standard Deviation
