# Supplementary material for: Evaluation of Change in Center of Pressure During Perturbation of Balance Including Blindfolding in Healthy Dogs
Source: Animals (Basel). 2025 Jun 18;15(12):1790. doi: 10.3390/ani15121790 (PMC12189757; doi:10.3390/ani15121790)
Supplement: Supplementary file 1 [file animals-15-01790-s001.zip › Supplementary F - Histograms.pdf]

Distributions Label=Blindfold

Area

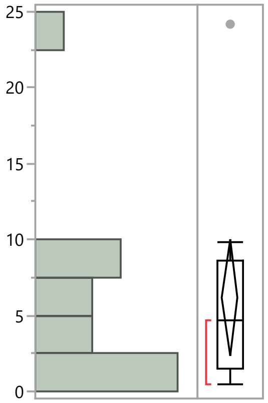

Quantiles

100.0%	maximum	24.1702
99.5%		24.1702
97.5%		24.1702
90.0%		18.445587
75.0%	quartile	8.62844
50.0%	median	4.71234
25.0%	quartile	1.51083
10.0%		0.6367
2.5%		0.4531
0.5%		0.4531
0.0%	minimum	0.4531

Summary Statistics

Mean	6.1942067
Std Dev	6.3170817
Std Err Mean	1.7520432
Upper 95% Mean	10.011581
Lower 95% Mean	2.3768324
N	13

Distance

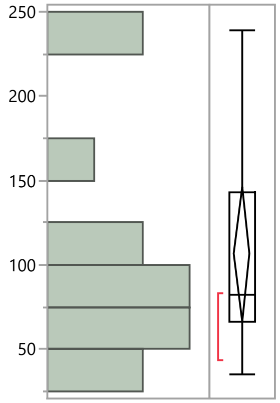

Quantiles

100.0%	maximum	238.68
99.5%		238.68
97.5%		238.68
90.0%		235.63467
75.0%	quartile	142.809
50.0%	median	82.208
25.0%	quartile	66.248333
10.0%		38.489
2.5%		34.895
0.5%		34.895
0.0%	minimum	34.895

Summary Statistics

Mean	106.50187
Std Dev	66.392127
Std Err Mean	18.413863
Upper 95% Mean	146.62223
Lower 95% Mean	66.381511
N	13

Variance

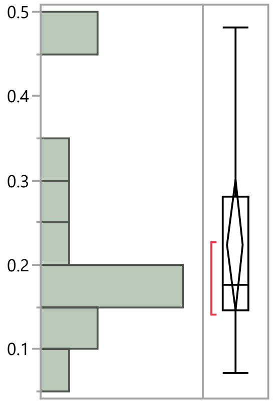

Quantiles

100.0%	maximum	0.4809667
99.5%		0.4809667
97.5%		0.4809667
90.0%		0.478508
75.0%	quartile	0.28026
50.0%	median	0.17632
25.0%	quartile	0.1458667
10.0%		0.0844772
2.5%		0.07251
0.5%		0.07251
0.0%	minimum	0.07251

Summary Statistics

Mean	0.2237737
Std Dev	0.1282559
Std Err Mean	0.0355718
Upper 95% Mean	0.301278
Lower 95% Mean	0.1462694
N	13

Cranial Caudal Distance

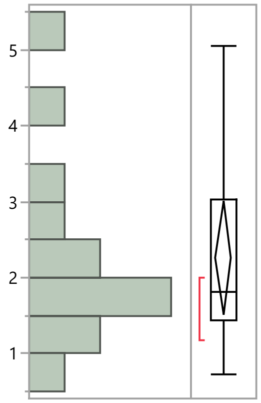

Quantiles

100.0%	maximum	5.0546667
99.5%		5.0546667
97.5%		5.0546667
90.0%		4.66712
75.0%	quartile	3.0341
50.0%	median	1.8199
25.0%	quartile	1.44084
10.0%		0.908996
2.5%		0.7281
0.5%		0.7281
0.0%	minimum	0.7281

Summary Statistics

Mean	2.2594959
Std Dev	1.2402226
Std Err Mean	0.3439759
Upper 95% Mean	3.0089549
Lower 95% Mean	1.5100369
N	13

Left Right Distance

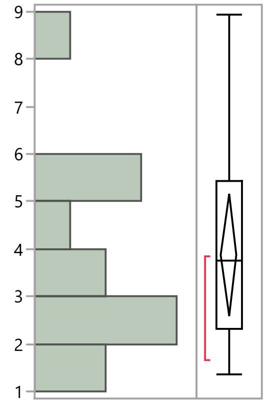

Quantiles

100.0%	maximum	8.9408
99.5%		8.9408
97.5%		8.9408
90.0%		7.65016
75.0%	quartile	5.4354667
50.0%	median	3.7576
25.0%	quartile	2.3179
10.0%		1.482135
2.5%		1.357025
0.5%		1.357025
0.0%	minimum	1.357025

Summary Statistics

Mean	3.8698547
Std Dev	2.130306
Std Err Mean	0.5908406
Upper 95% Mean	5.1571858
Lower 95% Mean	2.5825237
N	13

Distributions Label=Difference Blindfold

Area

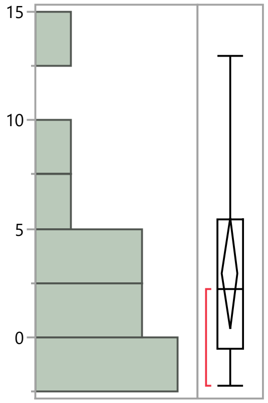

Quantiles

100.0%	maximum	12.96948
99.5%		12.96948
97.5%		12.96948
90.0%		10.790008
75.0%	quartile	5.46152
50.0%	median	2.22636
25.0%	quartile	-0.49419
10.0%		-1.934116
2.5%		-2.21994
0.5%		-2.21994
0.0%	minimum	-2.21994

Summary Statistics

Mean	2.9760205
Std Dev	4.224468
Std Err Mean	1.1716566
Upper 95% Mean	5.528841
Lower 95% Mean	0.4232
N	13

Distance

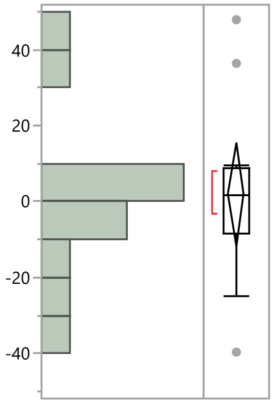

Quantiles

100.0%	maximum	47.86
99.5%		47.86
97.5%		47.86
90.0%		43.262667
75.0%	quartile	8.887
50.0%	median	1.742
25.0%	quartile	-8.537
10.0%		-33.689
2.5%		-39.595
0.5%		-39.595
0.0%	minimum	-39.595

Summary Statistics

Mean	1.9677179
Std Dev	22.618856
Std Err Mean	6.2733418
Upper 95% Mean	15.636156
Lower 95% Mean	-11.70072
N	13

Variance

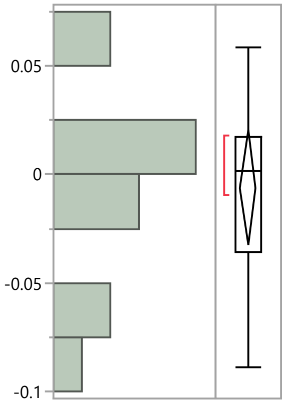

Quantiles

100.0%	maximum	0.05864667
99.5%		0.05864667
97.5%		0.05864667
90.0%		0.056804
75.0%	quartile	0.01745667
50.0%	median	0.0019
25.0%	quartile	-0.0359
10.0%		-0.0811908
2.5%		-0.08847
0.5%		-0.08847
0.0%	minimum	-0.08847

Summary Statistics

Mean	-0.006045
Std Dev	0.044225
Std Err Mean	0.0122658
Upper 95% Mean	0.0206802
Lower 95% Mean	-0.03277
N	13

Cranial Caudal Distance

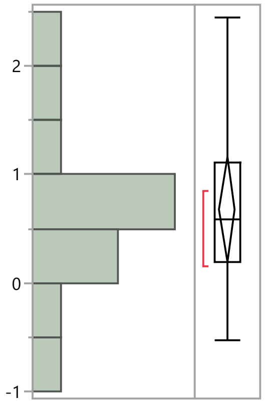

Quantiles

100.0%	maximum	2.4460667
99.5%		2.4460667
97.5%		2.4460667
90.0%		2.123528
75.0%	quartile	1.1071667
50.0%	median	0.58922
25.0%	quartile	0.19773
10.0%		-0.484084
2.5%		-0.51998
0.5%		-0.51998
0.0%	minimum	-0.51998

Summary Statistics

Mean	0.6810682
Std Dev	0.801795
Std Err Mean	0.2223779
Upper 95% Mean	1.1655881
Lower 95% Mean	0.1965483
N	13

Left Right Distance

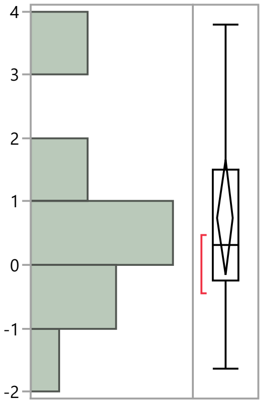

Quantiles

100.0%	maximum	3.7924
99.5%		3.7924
97.5%		3.7924
90.0%		3.6268533
75.0%	quartile	1.50901
50.0%	median	0.3126
25.0%	quartile	-0.244433
10.0%		-1.156705
2.5%		-1.628375
0.5%		-1.628375
0.0%	minimum	-1.628375

Summary Statistics

Mean	0.7466547
Std Dev	1.5104034
Std Err Mean	0.4189105
Upper 95% Mean	1.6593824
Lower 95% Mean	-0.166073
N	13

Distributions Label=Difference Headturn

Area

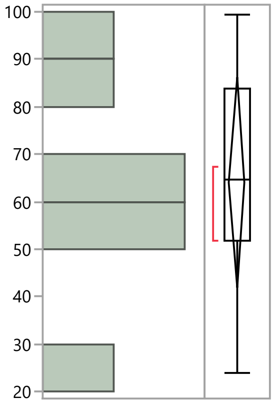

Quantiles

100.0%	maximum	99.46822
99.5%		99.46822
97.5%		99.46822
90.0%		99.46822
75.0%	quartile	83.83952
50.0%	median	64.5302
25.0%	quartile	51.72584
10.0%		24.06128
2.5%		24.06128
0.5%		24.06128
0.0%	minimum	24.06128

Summary Statistics

Mean	64.104183
Std Dev	23.961138
Std Err Mean	9.0564587
Upper 95% Mean	86.264539
Lower 95% Mean	41.943827
N	7

Distance

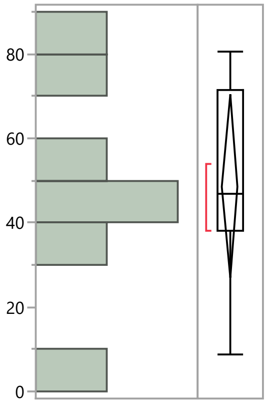

Quantiles

100.0%	maximum	80.58
99.5%		80.58
97.5%		80.58
90.0%		80.58
75.0%	quartile	71.457333
50.0%	median	46.864
25.0%	quartile	38.052
10.0%		8.814
2.5%		8.814
0.5%		8.814
0.0%	minimum	8.814

Summary Statistics

Mean	48.619619
Std Dev	23.563771
Std Err Mean	8.9062681
Upper 95% Mean	70.412472
Lower 95% Mean	26.826766
N	7

Variance

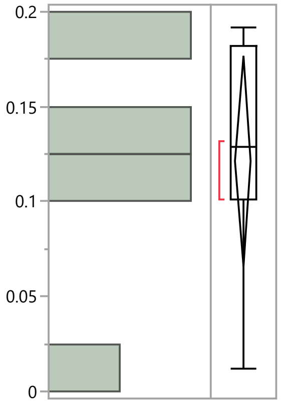

Quantiles

100.0%	maximum	0.19136
99.5%		0.19136
97.5%		0.19136
90.0%		0.19136
75.0%	quartile	0.18206
50.0%	median	0.1286
25.0%	quartile	0.10132
10.0%		0.01224
2.5%		0.01224
0.5%		0.01224
0.0%	minimum	0.01224

Summary Statistics

Mean	0.1212143
Std Dev	0.0597873
Std Err Mean	0.0225975
Upper 95% Mean	0.1765084
Lower 95% Mean	0.0659202
N	7

Cranial Caudal Distance

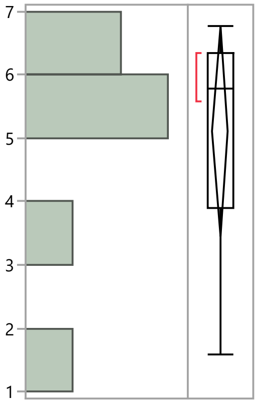

Quantiles

100.0%	maximum	6.76466
99.5%		6.76466
97.5%		6.76466
90.0%		6.76466
75.0%	quartile	6.35342
50.0%	median	5.7740533
25.0%	quartile	3.89772
10.0%		1.57952
2.5%		1.57952
0.5%		1.57952
0.0%	minimum	1.57952

Summary Statistics

Mean	5.1126248
Std Dev	1.7986287
Std Err Mean	0.6798178
Upper 95% Mean	6.7760789
Lower 95% Mean	3.4491706
N	7

Left Right Distance

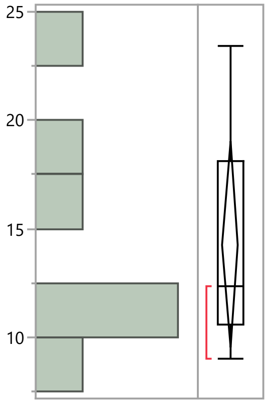

Quantiles

100.0%	maximum	23.396333
99.5%		23.396333
97.5%		23.396333
90.0%		23.396333
75.0%	quartile	18.098
50.0%	median	12.36
25.0%	quartile	10.5912
10.0%		9.0436
2.5%		9.0436
0.5%		9.0436
0.0%	minimum	9.0436

Summary Statistics

Mean	14.257762
Std Dev	5.1294629
Std Err Mean	1.9387548
Upper 95% Mean	19.001724
Lower 95% Mean	9.5137999
N	7

Distributions Label=Difference Perturbation

Area

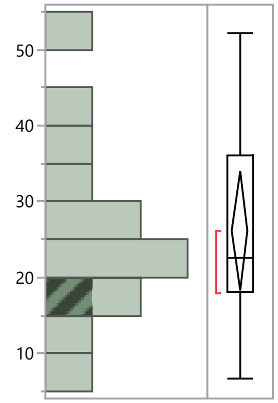

Quantiles

100.0%	maximum	52.2392
99.5%		52.2392
97.5%		52.2392
90.0%		49.02096
75.0%	quartile	36.069537
50.0%	median	22.57358
25.0%	quartile	18.0371
10.0%		8.437576
2.5%		6.77728
0.5%		6.77728
0.0%	minimum	6.77728

Summary Statistics

Mean	26.122132
Std Dev	12.923571
Std Err Mean	3.5843537
Upper 95% Mean	33.931768
Lower 95% Mean	18.312496
N	13

Distance

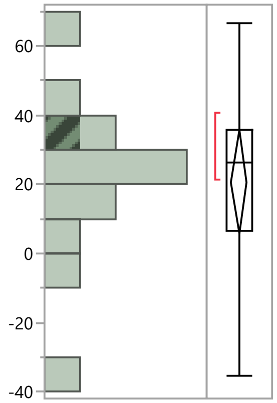

Quantiles

100.0%	maximum	66.61
99.5%		66.61
97.5%		66.61
90.0%		56.2796
75.0%	quartile	35.745
50.0%	median	26.244
25.0%	quartile	6.68
10.0%		-23.5008
2.5%		-35.36
0.5%		-35.36
0.0%	minimum	-35.36

Summary Statistics

Mean	20.655846
Std Dev	24.918953
Std Err Mean	6.911274
Upper 95% Mean	35.714219
Lower 95% Mean	5.5974737
N	13

Variance

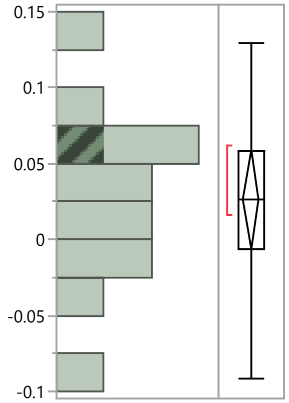

Quantiles

100.0%	maximum	0.12972
99.5%		0.12972
97.5%		0.12972
90.0%		0.108112
75.0%	quartile	0.05832
50.0%	median	0.0265
25.0%	quartile	-0.00616
10.0%		-0.065392
2.5%		-0.09168
0.5%		-0.09168
0.0%	minimum	-0.09168

Summary Statistics

Mean	0.0265728
Std Dev	0.0538933
Std Err Mean	0.0149473
Upper 95% Mean	0.0591402
Lower 95% Mean	-0.005995
N	13

Cranial Caudal Distance

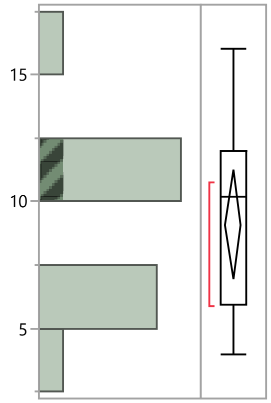

Quantiles

100.0%	maximum	16.01556
99.5%		16.01556
97.5%		16.01556
90.0%		14.561224
75.0%	quartile	11.98443
50.0%	median	10.22166
25.0%	quartile	5.91781
10.0%		4.488952
2.5%		3.98132
0.5%		3.98132
0.0%	minimum	3.98132

Summary Statistics

Mean	9.0860338
Std Dev	3.5847487
Std Err Mean	0.9942304
Upper 95% Mean	11.252276
Lower 95% Mean	6.9197919
N	13

Left Right Distance

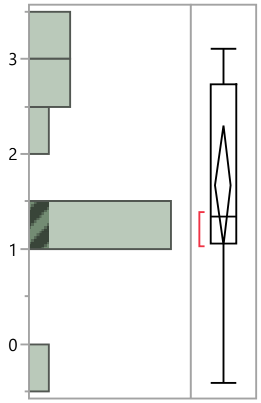

Quantiles

100.0%	maximum	3.1086
99.5%		3.1086
97.5%		3.1086
90.0%		3.08372
75.0%	quartile	2.7344
50.0%	median	1.3464
25.0%	quartile	1.0622
10.0%		0.16336
2.5%		-0.4148
0.5%		-0.4148
0.0%	minimum	-0.4148

Summary Statistics

Mean	1.673
Std Dev	1.0446281
Std Err Mean	0.2897277
Upper 95% Mean	2.3042624
Lower 95% Mean	1.0417376
N	13

Distributions Label=Headturn

Area

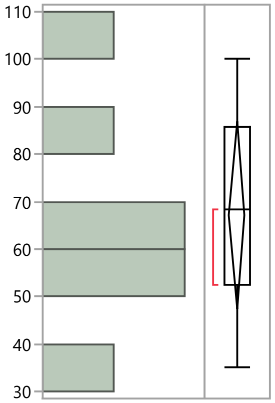

Quantiles

100.0%	maximum	100.11
99.5%		100.11
97.5%		100.11
90.0%		100.11
75.0%	quartile	85.798
50.0%	median	68.432
25.0%	quartile	52.586
10.0%		35.262
2.5%		35.262
0.5%		35.262
0.0%	minimum	35.262

Summary Statistics

Mean	67.056
Std Dev	21.360429
Std Err Mean	8.0734832
Upper 95% Mean	86.811102
Lower 95% Mean	47.300898
N	7

Distance

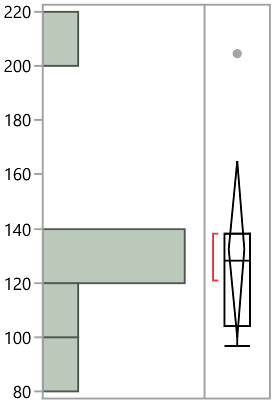

Quantiles

100.0%	maximum	204.5
99.5%		204.5
97.5%		204.5
90.0%		204.5
75.0%	quartile	138
50.0%	median	128.32
25.0%	quartile	104.004
10.0%		97.09
2.5%		97.09
0.5%		97.09
0.0%	minimum	97.09

Summary Statistics

Mean	132.36105
Std Dev	35.193447
Std Err Mean	13.301873
Upper 95% Mean	164.90956
Lower 95% Mean	99.812538
N	7

Variance

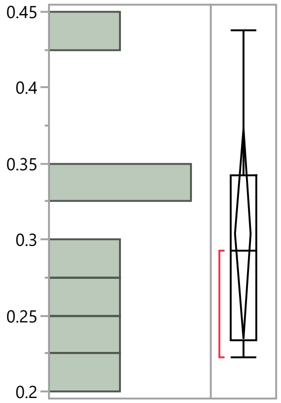

Quantiles

100.0%	maximum	0.43762
99.5%		0.43762
97.5%		0.43762
90.0%		0.43762
75.0%	quartile	0.3423
50.0%	median	0.2925
25.0%	quartile	0.2335
10.0%		0.22242
2.5%		0.22242
0.5%		0.22242
0.0%	minimum	0.22242

Summary Statistics

Mean	0.3039057
Std Dev	0.0747155
Std Err Mean	0.0282398
Upper 95% Mean	0.373006
Lower 95% Mean	0.2348054
N	7

Cranial Caudal Distance

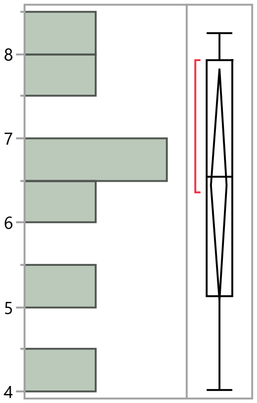

Quantiles

100.0%	maximum	8.2516
99.5%		8.2516
97.5%		8.2516
90.0%		8.2516
75.0%	quartile	7.923
50.0%	median	6.5443333
25.0%	quartile	5.1284
10.0%		4.0256
2.5%		4.0256
0.5%		4.0256
0.0%	minimum	4.0256

Summary Statistics

Mean	6.4515048
Std Dev	1.4884795
Std Err Mean	0.5625924
Upper 95% Mean	7.8281187
Lower 95% Mean	5.0748908
N	7

Left Right Distance

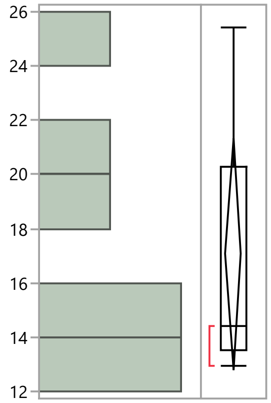

Quantiles

100.0%	maximum	25.403333
99.5%		25.403333
97.5%		25.403333
90.0%		25.403333
75.0%	quartile	20.286
50.0%	median	14.4068
25.0%	quartile	13.526
10.0%		12.9454
2.5%		12.9454
0.5%		12.9454
0.0%	minimum	12.9454

Summary Statistics

Mean	17.063933
Std Dev	4.6071059
Std Err Mean	1.7413223
Upper 95% Mean	21.324796
Lower 95% Mean	12.803071
N	7

Distributions Label=Perturbation

Area

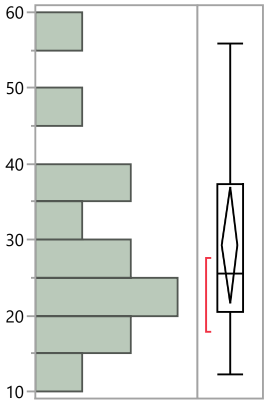

Quantiles

100.0%	maximum	55.846
99.5%		55.846
97.5%		55.846
90.0%		53.3708
75.0%	quartile	37.369667
50.0%	median	25.478
25.0%	quartile	20.5025
10.0%		14.52668
2.5%		12.2258
0.5%		12.2258
0.0%	minimum	12.2258

Summary Statistics

Mean	29.340318
Std Dev	12.64036
Std Err Mean	3.505805
Upper 95% Mean	36.978811
Lower 95% Mean	21.701825
N	13

Distance

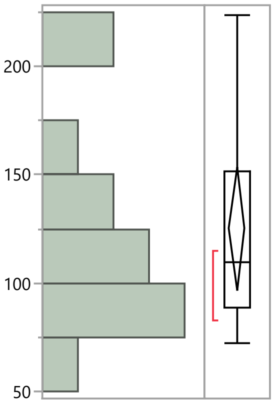

Quantiles

100.0%	maximum	223.62
99.5%		223.62
97.5%		223.62
90.0%		216.508
75.0%	quartile	151.84
50.0%	median	109.7
25.0%	quartile	88.949
10.0%		76.4548
2.5%		72.074
0.5%		72.074
0.0%	minimum	72.074

Summary Statistics

Mean	125.19
Std Dev	47.238405
Std Err Mean	13.101576
Upper 95% Mean	153.73588
Lower 95% Mean	96.644117
N	13

Variance

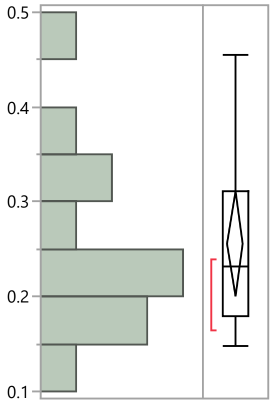

Quantiles

100.0%	maximum	0.4553
99.5%		0.4553
97.5%		0.4553
90.0%		0.430244
75.0%	quartile	0.31193
50.0%	median	0.23186
25.0%	quartile	0.1795933
10.0%		0.155228
2.5%		0.1485
0.5%		0.1485
0.0%	minimum	0.1485

Summary Statistics

Mean	0.2563913
Std Dev	0.0913972
Std Err Mean	0.025349
Upper 95% Mean	0.3116221
Lower 95% Mean	0.2011605
N	13

Cranial Caudal Distance

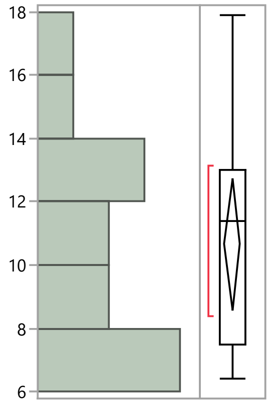

Quantiles

100.0%	maximum	17.92
99.5%		17.92
97.5%		17.92
90.0%		16.3936
75.0%	quartile	13.02
50.0%	median	11.38
25.0%	quartile	7.4813
10.0%		6.54996
2.5%		6.4274
0.5%		6.4274
0.0%	minimum	6.4274

Summary Statistics

Mean	10.664462
Std Dev	3.4504606
Std Err Mean	0.9569856
Upper 95% Mean	12.749554
Lower 95% Mean	8.5793691
N	13

Left Right Distance

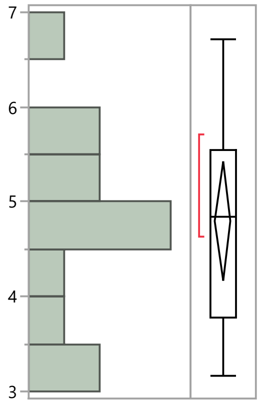

Quantiles

100.0%	maximum	6.7072
99.5%		6.7072
97.5%		6.7072
90.0%		6.3668
75.0%	quartile	5.5452
50.0%	median	4.8356
25.0%	quartile	3.7801
10.0%		3.27468
2.5%		3.173
0.5%		3.173
0.0%	minimum	3.173

Summary Statistics

Mean	4.7962
Std Dev	1.0421835
Std Err Mean	0.2890497
Upper 95% Mean	5.4259852
Lower 95% Mean	4.1664148
N	13

Distributions Label=Standing

Area

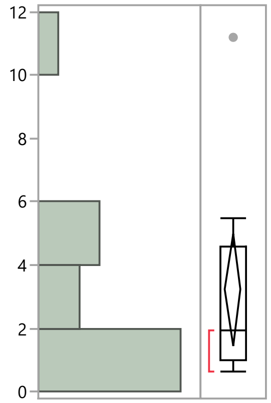

Quantiles

100.0%	maximum	11.20072
99.5%		11.20072
97.5%		11.20072
90.0%		8.906192
75.0%	quartile	4.6082
50.0%	median	1.95848
25.0%	quartile	1.00429
10.0%		0.705868
2.5%		0.64178
0.5%		0.64178
0.0%	minimum	0.64178

Summary Statistics

Mean	3.2181862
Std Dev	2.9371612
Std Err Mean	0.814622
Upper 95% Mean	4.9930949
Lower 95% Mean	1.4432774
N	13

Distance

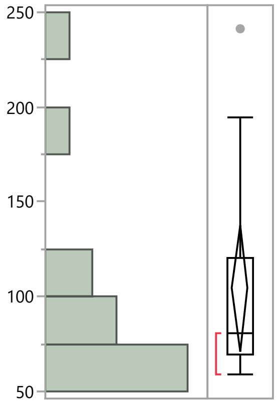

Quantiles

100.0%	maximum	241.2
99.5%		241.2
97.5%		241.2
90.0%		222.6
75.0%	quartile	120.464
50.0%	median	80.466
25.0%	quartile	69.69
10.0%		60.2932
2.5%		59.038
0.5%		59.038
0.0%	minimum	59.038

Summary Statistics

Mean	104.53415
Std Dev	54.909413
Std Err Mean	15.229131
Upper 95% Mean	137.71558
Lower 95% Mean	71.352728
N	13

Variance

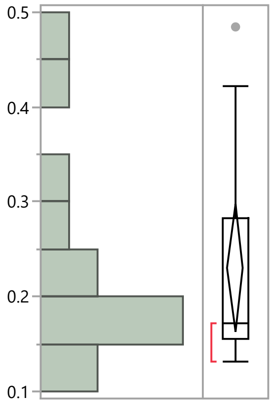

Quantiles

100.0%	maximum	0.48434
99.5%		0.48434
97.5%		0.48434
90.0%		0.459532
75.0%	quartile	0.28354
50.0%	median	0.1727
25.0%	quartile	0.15596
10.0%		0.13858
2.5%		0.1321
0.5%		0.1321
0.0%	minimum	0.1321

Summary Statistics

Mean	0.2298185
Std Dev	0.1112691
Std Err Mean	0.0308605
Upper 95% Mean	0.2970577
Lower 95% Mean	0.1625792
N	13

Cranial Caudal Distance

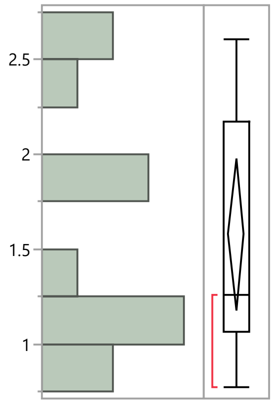

Quantiles

100.0%	maximum	2.6086
99.5%		2.6086
97.5%		2.6086
90.0%		2.59724
75.0%	quartile	2.17526
50.0%	median	1.25934
25.0%	quartile	1.06326
10.0%		0.770608
2.5%		0.77028
0.5%		0.77028
0.0%	minimum	0.77028

Summary Statistics

Mean	1.5784277
Std Dev	0.6644858
Std Err Mean	0.1842952
Upper 95% Mean	1.9799725
Lower 95% Mean	1.1768829
N	13

Left Right Distance

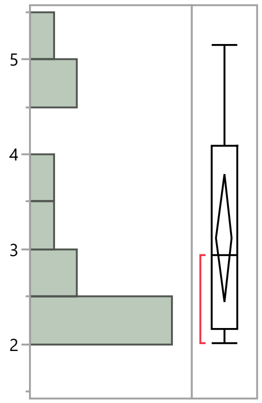

Quantiles

100.0%	maximum	5.1484
99.5%		5.1484
97.5%		5.1484
90.0%		5.01816
75.0%	quartile	4.0875
50.0%	median	2.9348
25.0%	quartile	2.1535
10.0%		2.02292
2.5%		2.007
0.5%		2.007
0.0%	minimum	2.007

Summary Statistics

Mean	3.1232
Std Dev	1.1152782
Std Err Mean	0.3093225
Upper 95% Mean	3.7971559
Lower 95% Mean	2.4492441
N	13
